# Males Under-Estimate Academic Performance of Their Female Peers in Undergraduate Biology Classrooms

**DOI:** 10.1371/journal.pone.0148405

**Published:** 2016-02-10

**Authors:** Daniel Z. Grunspan, Sarah L. Eddy, Sara E. Brownell, Benjamin L. Wiggins, Alison J. Crowe, Steven M. Goodreau

**Affiliations:** 1 Department of Anthropology, University of Washington, Seattle, WA, 98185, United States of America; 2 Texas Institute for Discovery Education in Science, College of Natural Sciences, University of Texas at Austin, Austin, TX, 78712, United States of America; 3 School of Life Sciences, Arizona State University, Tempe, AZ, 85287, United States of America; 4 Department of Biology, University of Washington, Seattle, WA, 98195, United States of America; University of Missouri, UNITED STATES

## Abstract

Women who start college in one of the natural or physical sciences leave in greater proportions than their male peers. The reasons for this difference are complex, and one possible contributing factor is the social environment women experience in the classroom. Using social network analysis, we explore how gender influences the confidence that college-level biology students have in each other’s mastery of biology. Results reveal that males are more likely than females to be named by peers as being knowledgeable about the course content. This effect increases as the term progresses, and persists even after controlling for class performance and outspokenness. The bias in nominations is specifically due to males over-nominating their male peers relative to their performance. The over-nomination of male peers is commensurate with an overestimation of male grades by 0.57 points on a 4 point grade scale, indicating a strong male bias among males when assessing their classmates. Females, in contrast, nominated equitably based on student performance rather than gender, suggesting they lacked gender biases in filling out these surveys. These trends persist across eleven surveys taken in three different iterations of the same Biology course. In every class, the most renowned students are always male. This favoring of males by peers could influence student self-confidence, and thus persistence in this STEM discipline.

## Introduction

Male faculty members outnumber female faculty members in every science, technology, engineering, and math (STEM) discipline [1]. The attrition of female STEM students from their disciplines can be seen in early stages of the progression to STEM careers including the transition into college and graduate school [2]. The experiences of women in STEM that may lead to this attrition can be subtle. It is generally no longer considered a matter of outright discrimination, but rather the accumulation of smaller experiences that determine whether a female student identifies with and persists in a scientific field [3–6]. One factor linked to persistence in STEM fields is self-confidence. This factor is heavily influenced by social interactions, particularly for women in historically male-dominated fields [7–9]. For example, a student whose abilities are endorsed by an influential person may experience increased performance and confidence; conversely, a student not receiving this affirmation experiences a decrease in performance and confidence [10,11]. While formative experiences like these can occur throughout one’s life, certain periods are more influential than others. Individuals are particularly attuned to cues confirming or discrediting their ability to succeed in a field during major transition periods [8,12]. Student experiences at one of those key transition points, introductory STEM courses, thus seem likely to influence current and future decisions to persist in STEM disciplines.

STEM faculty members provide some of the first professional feedback and interactions that students receive in their disciplines. Unfortunately, both male and female faculty members behave in ways that subtly favor males in STEM disciplines: (a) they are more likely to spend time mentoring males [13], (b) they are more likely to respond to emails from males [14], and (c) they are more likely to call on males in class [15]. These subtle yet consistent biases appear to cause at least some female STEM students to experience a lower sense of belonging or confidence in their discipline, resulting in an increased tendency to leave science [16].

In addition to interactions with faculty members, interactions with other students could impact a student’s sense of belonging and confidence in her discipline. In contrast to the work on gender biases among faculty, only limited research has been performed on the disposition of current college-age students (the “millennial” generation) towards women in STEM and how this disposition may impact their female peers (but see [17,18]). Such research would not only help us to measure one force that may be acting to decrease undergraduate females’ sense of belonging in STEM fields; it would also help us predict whether we can expect these implicit biases to persist in future STEM faculty.

In this paper we focus on the formative experience of nascent STEM professionals during an introductory college science course, a key transition period for the development of a STEM identity [19]. We explore this question in a biology classroom. We chose this field because females and males enroll equally in this discipline at the undergraduate level [20] and thus should represent a conservative case of the biases women in introductory STEM courses experience.

We explore the impact of gender on how students perceive their peers, as well as how students are perceived by their peers. It is important to note that the gender data used in this study come from the school registrar, and are thus defined by information given during student enrollment. The registrar constrains choice for gender identification to ‘male’ or ‘female’ choices. Given these complications, we choose to refer to student genders, but recognize that in some cases the data may not accurately reflect the true gender identity of each student.

To investigate how gender impacts peer perception, undergraduate students were asked to anonymously list class peers who they felt were “strong in their understanding of classroom material” at multiple time points throughout three iterations of a large introductory biology class. We employ longitudinal social network analyses of these data to (1) describe the distribution of nominations received between males and females, and (2) identify the factors that predict who a student will nominate as having mastered the content in their field. Finally, (3) we examine the characteristics of students receiving the most nominations in each class (to whom we refer to as “celebrities”). We focus on these students given our assumption that their ability to draw widespread acknowledgment of their excellence makes them among the most likely in the class to continue in the field beyond the undergraduate level.

## Materials and Methods

### Ethics statement

We obtained human subjects approval from the University of Washington Institutional Review Board (#44438). Because students were not asked to do anything outside of the normal class curriculum, an altered consent process was approved for use in this study. Subjects were informed that a research study was taking place and that their data would be analyzed as part of this study. Students were informed that they could opt out of the study at any time by filling out a form in a centralized office.

### Classroom and classroom data

Data come from three different iterations of the same large introductory undergraduate biology class (*n = 196*, *759*, *and 760*, *corresponding to class A*, *B*, *and C*, *respectively*) at a large American university that engages in very high research activity (an R1 university). The class of interest is the second in a series of three introductory Biology classes, where the first class in the series served as a pre-requisite. Because this was the second class, many students enter the class already knowing many of their peers. Student demographic information, including gender, was collected from the Office of the University Registrar and course grades from the Department of Biology.

All three iterations of this course included a lab section with a maximum capacity of 24 students that met once a week for several hours. Classes A, B, and C contained 9, 33, and 33 lab sections, respectively. The gender distribution within lab sections is approximately normal and mirrors that of the overall class (Mean = 57.4% female, SD = 0.11). The lecture portion of the course met for 50 minutes a day four days out of the week, and employed active learning techniques in all three iterations of the course. In all three cases, lectures were split into two sections with approximately 100 students in each for class A, and approximately 375 in each for Classes B and C; the instructor stayed consistent between lectures each class day to assure minimal differences between the two sections. Classes A and B were both taught by a male instructor, while Class C had three total instructors: two male instructors teaching 75% of class days and one female instructor teaching 25% of class days. All three iterations of the class included three exams spaced throughout the quarter, and a non-cumulative final exam that took place one week after the end of the quarter. Grades were not publically posted in any of the three classes.

A measurement of student outspokenness was collected by polling the course instructor of record immediately after the end of each course, and thus represents active participation as perceived by the instructor who was blind to the hypotheses being tested. Thus, a student who frequently offers an incorrect answer in class is considered equally outspoken as students who frequently offer the correct answers. Because measurements come from instructors, the list may be subject to each instructor’s own implicit biases.

All three classes consisted primarily of white and Asian students (40.5% and 29.9% of entire population across the three classes, respectively). Student ethnicity is not included in these analyses for two reasons. First, the diversity in each classroom is such that statistical power to understand the perception of minority students is lacking. Second, this issue is substantial enough to warrant its own separate analysis.

### Student networks

All network surveys were administered via a confidential online survey. For Class A, students were given a class roster after the first and second exams and were asked to mark students they felt were particularly strong with class material. In Class B students were asked at the beginning of the class to list students by name who they felt would do particularly well in the course. After the first, second, and third exams, they were asked to list students they felt were particularly strong with class material. The same collection method was performed in Class C as Class B, but in addition students were surveyed again before the final exam of the course. Surveys in Class C distinguished between students who responded and didn’t know anyone they felt were knowledgeable and students who didn’t list anybody due to a non-response to the survey. Thus, Class C offers the most accurate means to calculate response rates. An average of 81.4% (SD = 0.02) of students responded across the five surveys in this class, with 82.8% of female students responding (SD = 0.02) and 79.9% of males responding (SD = 0.01). We have no reason to believe that Classes A or B differed in response rates, or that response rates were skewed by gender in any manner.

### Analysis of nominations

To assess the hypotheses about nomination structure, we used exponential-family random graph models (ERGMs). This approach can be thought of as a kind of generalization of logistic regression to social networks–with the log-odds of a tie (here, a nomination) between two actors being dependent on a set of predictors of interest [21]. Those predictors may include characteristics of either or both nodes (e.g. their gender, class performance or outspokenness). However, it can also include structural factors involving the other ties in the network–e.g. the tendency for ties in a directed network to be mutual, or to form a triangle. When such structural terms are present, ties become conditionally dependent and estimation becomes more difficult, with Markov chain Monte Carlo-based methods the current state of the art for estimation [22]. Nevertheless, the coefficients may still be interpreted in terms of their contribution to the conditional log-odds of a tie, given all of the other ties in the network.

We specify two models, both of the general form:

$$\text{logit}\;\left(Y_{ij}=1\;|y_{ij}^{c}\right)=\sum_{k=1}^{n}\;\theta_{k}\;\delta_{k}$$

where *Y*_*ij*_ represents the value of the tie from *i* to *j*, which equals 1 if *i* nominates *j* and 0 if they did not (we discuss missing data for these values in the SI). The quantity $y_{ij}^{c}$ represents the complement of *y*_*ij*,_ i.e. the state of all of the ties in the network other than *y*_*ij*._ The δ vector represents the amount by which the model statistics change when *y*_*ij*_ is toggled from 0 to 1, and the θ vector represents the coefficients on these statistics.

The first model contains seven model statistics (δ_1_ through δ_7_) and the second model contains nine (δ_1_ through δ_9_):

δ _1_ = 1 for all dyads [the main effect or intercept];
δ _2_ = 1 if *j* nominates *i*, and 0 otherwise [mutuality];
δ _3_ = 1 if *i* is female and 0 otherwise [female nominator];
δ _4_ = 1 if both *i* and *j* are female and 0 otherwise [female-female bias];
δ _5_ = 1 if both *i* and *j* are male and 0 otherwise [male-male bias];
δ _6_ = 1 if *i* and *j* are in the same lab section [lab homophily];
δ _7_ = -1 if *j* has no nominations other than that from *i* [0-indegree];
δ _8_ = j’s final grade in the class [grade of nominee];
δ _9_ = 1 if *j* is outspoken, and 0 otherwise [outspokenness of nominee];

We use the R package *network* to process and store the data, and the R package *ergm* to estimate the θ coefficients for our two models for each survey wave [22,23]. The terms involving gender, grade, or outspokenness represent our core theoretical measures. We include mutuality since it is a basic phenomenon in directed networks (those where the relationship from *i* to *j* does not necessarily equal that from *j* to *i*), and we include lab homophily given that labs are a major structural element of the course. We include a unique propensity for individuals to have no nominations (called 0-indegree in network terminology) since this dramatically improved the fit of the model to the observed in-degree distribution, which is a condition for the statistical inference we later conduct (see S1 appendix for more information). Moreover, it is reasonable to expect that measures of renown such as that here would have more variation than expected by chance–that is, with more students who have either no or many nominations than otherwise expected. The δ on this term is negative given the unique condition that adding a tie reduces the statistic of interest (nodes without ties).

## Results

### Classroom data and student outspokenness

A summary of student data stratified by gender can be found in Table 1. All three classes consisted of a numerical female majority; classes A, B, and C were 56%, 55.4%, and 58.4% female, respectively. Males averaged higher course grades than females in all three classes; the differences in grade in class C, but not A and B, is significant (p = 0.0171; unpaired t-test).

Proportionately, more males than females were listed as outspoken (p = 0.0258; Mantel-Haenszel test). While instructor bias causing this gender difference in outspoken status is something we cannot check, it is worth noting that any male bias in the assignment of outspoken status would make our estimates of male bias in peer perception more conservative than they actually are.

**Table 1:** Student demographics from all three classes. Classes are majority female in all three cases. Males performed slightly better than females in each class, and also tended to be more outspoken. Numerical counts are accompanied by total percentage in the class in parentheses. Means are accompanied by standard deviations in parentheses.

	Class A		Class B		Class C	
	*Female*	*Male*	*Female*	*Male*	*Female*	*Male*
Total students	110 (56%)	86 (44%)	431 (55.4%)	328 (44.6%)	444 (58.4%)	316 (41.6%)
Mean class grade (out of 4.0)	2.68 (1.01)	2.93 (0.82)	2.74 (0.83)	2.86 (0.84)	2.75 (0.82)	2.89 (0.76)
Number of students listed as outspoken	16 (14.5%)	16 (16.3%)	64 (14.8%)	52 (15.8%)	98 (22.1%)	95 (30.1%)
Mean number nominations at S_1_	-	-	1.14 (1.50)	1.20 (1.73)	1.19 (1.52)	1.13 (1.52)
Mean number nominations at S_2_	1.05 (1.39)	1.60 (2.81)	0.98 (1.45)	1.16 (2.25)	1.01 (1.41)	1.08 (1.58)
Mean number nominations at S_3_	1.06 (1.55)	1.69 (2.95)	1.22 (1.55)	1.48 (2.44)	1.02 (1.43)	1.17 (1.78)
Mean number nominations at S_4_	-	-	1.12 (1.64)	1.55 (3.63)	1.23 (1.60)	1.44 (1.92)
Mean number nominations at S_5_	-	-	-	-	1.21 (1.55)	1.36 (1.87)

### Males are over-nominated by peers as mastering biology

Across the 11 peer perception surveys, students received an average of 1.20 nominations with a standard deviation of 1.85; males averaged 1.31 nominations with a standard deviation of 2.23, while females averaged 1.12 nominations with a standard deviation of 1.51. Males consistently received more nominations than females in every survey, with the first survey in Class C as the only exception.

**Fig 1:**
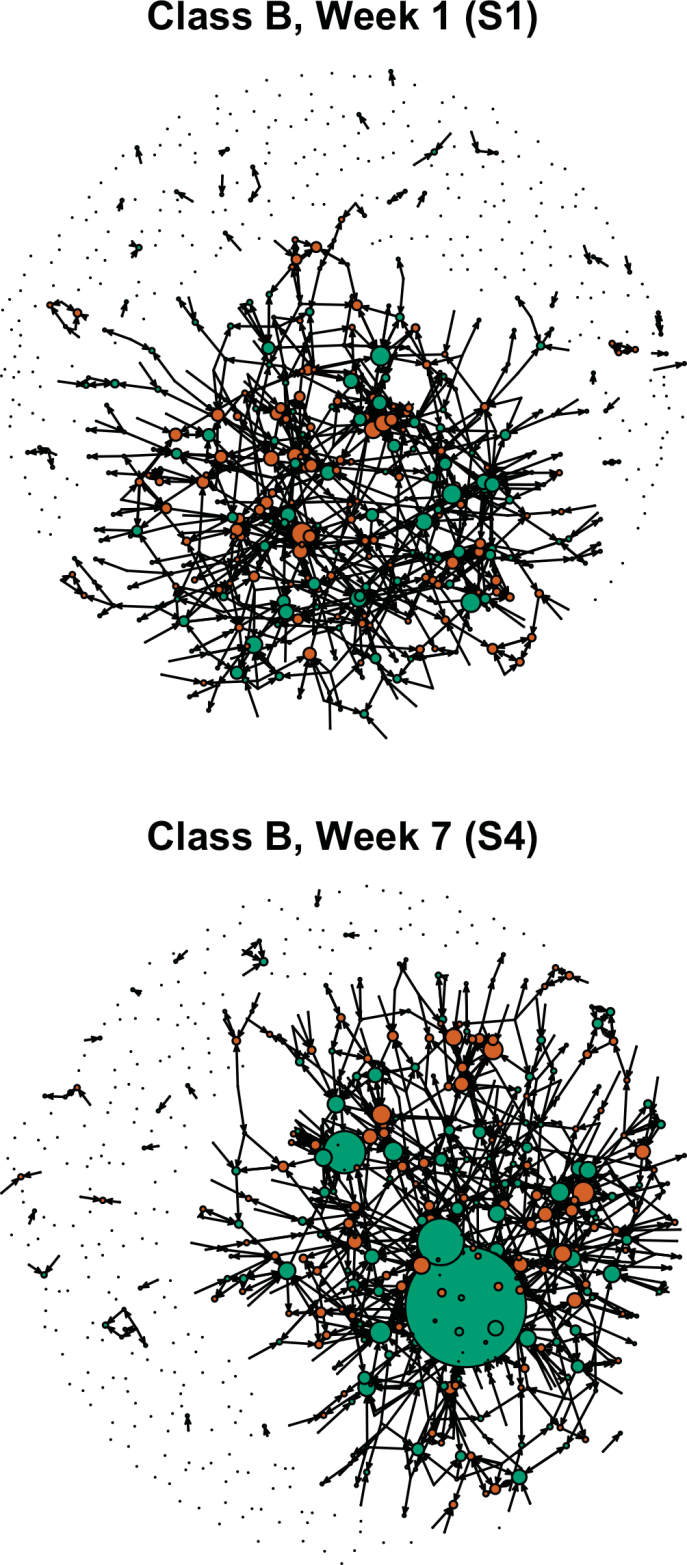
Unequal distribution of peer perception of mastery of content among genders grows over the term. Sociographs at the beginning of course (S1) and after exam 3 (S4) in class B. Male students are represented by green circles and females by orange circles. The size of nodes correlates with how many nominations each student received. Arrows show direction from the nominator to the nominee.

In all three classes, the number of nominations given to males increased throughout the course. This pattern was particularly strong in Classes B and C, where data were collected across a longer time span. No consistent longitudinal trend for females is visible in any of the three classes. Combined, these patterns result in a growing gender gap in the number of nominations received between males and females when comparing data from surveys early in the class to those taken later in the class (Fig 1; S1, S2 and S3 Figs).

To determine the significance of these results, we use Exponential Random Graph Models (ERGM). Our base model does not include grade or outspokenness in order to give an absolute sense of the gender differences in receiving nominations (S1 Table). In all 11 surveys, males show a significant bias toward nominating other males, with an absolute value greater than seen among females. In the last survey from Class A, females also show a bias towards nominating males, but show no significant bias either way in the remaining 10 surveys.

### Class performance and outspokenness predict classroom wide recognition, but males still nominate more males after controlling for these

Over-representation of males in received nominations could be explained either by the higher frequency of outspokenness in males, or the higher average grades achieved by males compared to females, as both of these measures may indicate that, on average, males indeed know the material better or at least make their knowledge more visible to their peers. To test these explanations, we expanded our ERGM to include the class grade and outspokenness of the nominee as mediating factors (Table 2).

**Table 2:** Coefficients from exponential random graph models from all 11 networks across all three courses, demonstrating that female bias towards nominating other females is not significant in any survey, while male bias towards nominating males is significant in all 11 surveys. Each column represents coefficients from a different survey (S): S1 surveys were taken the first week, S2 after the first exam, S3 after the second exam, and so on. Coefficients represent the influence on the log-odds of a nomination for each predictor; each is formally defined in the Methods section. Bolded coefficients indicate significance at α of 0.05. Positive coefficients indicate that ties are more likely to occur, while negative coefficients indicate that ties are less likely to occur. Values in parentheses represent 95% confidence intervals.

Coefficient name	Course A		Course B				Course C				
	S2	S3	S1	S2	S3	S4	S1	S2	S3	S4	S5
Intercept	**-7.42 (-8.18, -6.67)**	**-7.14 (-7.89, -6.40)**	**-8.27 (-8.66, -7.88)**	**-9.25 (-9.71, -8.78)**	**-8.86 (-9.26, -8.47)**	**-9.41 (-9.88, -8.93)**	**-8.11 (-8.51, -7.70)**	**-8.27 (-8.72, -7.82)**	**-8.26 (-8.68, -7.84)**	**-8.05 (-8.43, -7.66)**	**-8.18 (-8.56, -7.79)**
Mutuality	**3.66 (3.16, 4.15)**	**4.43 (3.94, 4.92)**	**5.60 (5.33, 5.86)**	**5.52 (5.22, 5.81)**	**5.70 (5.45, 5.95)**	**5.50 (5.21, 5.78)**	**5.81 (5.50, 6.12)**	**5.39 (5.09, 5.70)**	**5.47 (5.22, 5.72)**	**5.26 (4.98, 5.53)**	**5.46 (5.19, 5.72)**
Grade of nominee	**0.38 (0.19, 0.58)**	**0.33 (0.15, 0.52)**	**0.48 (0.38, 0.58)**	**0.74 (0.62, 0.86)**	**0.59 (0.49, 0.69)**	**0.77 (0.65, 0.89)**	**0.49 (0.39, 0.60)**	**0.53 (0.42, 0.64)**	**0.53 (0.42, 0.64)**	**0.51 (0.40, 0.62)**	**0.51 (0.41, 0.61)**
Outspokenness of nominee	**0.87 (0.59, 1.15)**	**0.67 (0.42, 0.92)**	**0.25 (0.12, 0.38)**	**0.49 (0.34, 0.64)**	**0.53 (0.39, 0.68)**	**0.64 (0.50, 0.77)**	0.02 (-0.11, 0.15)	**0.14 (0.00, 0.27)**	**0.15 (0.02, 0.28)**	**0.20 (0.07, 0.32)**	**0.15 (0.03, 0.27)**
Homophily on lab section	**1.44 (1.20, 1.68)**	**1.06 (0.82, 1.30)**	**0.73 (0.54, 0.92)**	**1.08 (0.91, 1.25)**	**1.20 (1.10, 1.29)**	**1.20 (1.05, 1.35)**	**1.12 (0.90, 1.34)**	**1.20 (1.01, 1.39)**	**1.29 (1.10, 1.47)**	**1.41 (1.21, 1.61)**	**1.41 (1.20, 1.62)**
0-indegree	**0.74 (0.23, 1.25)**	**1.31 (0.80, 1.83)**	**1.44 (1.18, 1.70)**	**1.20 (0.93, 1.48)**	**1.00 (0.75, 1.26)**	**1.31 (1.03, 1.60)**	**1.29 (0.99, 1.59)**	**1.30 (0.99, 1.60)**	**1.45 (1.16, 1.74)**	**1.25 (0.97, 1.53)**	**1.20 (0.91, 1.50)**
Female nominator	**0.84 (0.39, 1.28)**	**1.13 (0.67, 1.60)**	**0.53 (0.30, 0.77)**	**0.43 (0.19, 0.67)**	**0.59 (0.37, 0.81)**	**0.71 (0.50, 0.92)**	**0.25 (0.02, 0.48)**	**0.27 (0.03, 0.51)**	**0.29 (0.05, 0.54)**	0.16 (-0.05, 0.37)	**0.24 (0.00, 0.47)**
Female-female bias	-0.04 (-0.33, 0.25)	-0.21 (-0.49, 0.07)	0.08 (-0.06, 0.22)	0.01 (-0.15, 0.18)	-0.04 (-0.20, 0.11)	-0.11 (-0.26, 0.03)	0.14 (-0.04, 0.32)	0.12 (-0.05, 0.29)	0.09 (-0.07, 0.26)	0.11 (-0.04, 0.26)	0.15 (0.00, 0.31)
Male-male bias	**0.57 (0.15, 0.99)**	**0.59 (0.17, 1.02)**	**0.33 (0.11, 0.56)**	**0.25 (0.02, 0.48)**	**0.41 (0.22, 0.59)**	**0.46 (0.26, 0.67)**	**0.26 (0.04, 0.49)**	**0.29 (0.06, 0.51)**	**0.30 (0.09, 0.51)**	**0.28 (0.10, 0.46)**	**0.32 (0.14, 0.51)**

Cell entry = Point estimate (95% CI); S = survey number; Bold = significant (p < 0.05).

Note: for Course C Survey S5, the lower bound of the confidence interval is positive for female nominator and negative for female-female bias, although both round to 0 with only two decimal places.

Performance is a strong and significant predictor of receiving a nomination in every survey, indicating that students have an accurate sense of other students’ performance, despite not having any public way to view their peers’ grades. In addition, outspokenness has a significant effect in all but one case, indicating that students also nominate based on this trait. Being in the same lab section is also universally predictive of a nomination from one student to another. There is a significant tendency for there to be more students with no nominations than expected by chance given the overall nomination rates and the other terms in the model. The female nominator coefficient indicates that females make more nominations overall than males do, without considering the gender of those they nominate.

**Fig 2:**
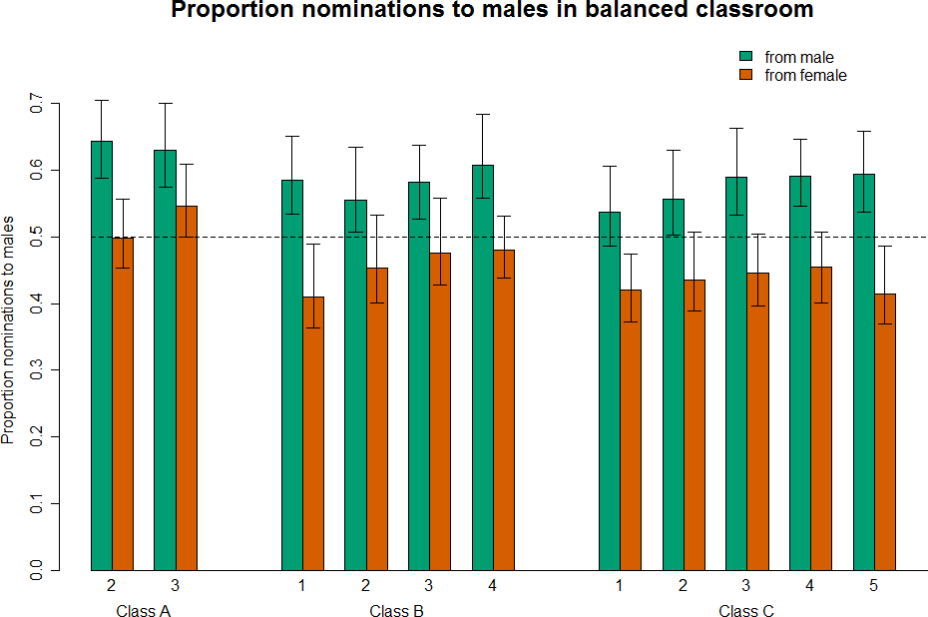
Males over-nominate males; females are closer to equitable in their nominations. Model based predictions for a hypothetical class comprising 50% males and 50% females. To isolate the effect of gender bias this class was also modeled as having an equal grade distribution and level of outspokenness across genders. We plot the results from 100 simulations for each of the models; the main bars represent the mean, and the whiskers reflect the range in which the central 95% of the simulations fall. Even with equal performance and outspokenness in this hypothetical class across all three model predictions, the longitudinal increase in bias of male students to nominate males remains. Female students also demonstrate a pattern of moving from female to male nominations over the course of each class.

With performance and outspokenness in the model, females no longer show a bias toward nominating males in any of the 11 surveys; their nominations do not diverge from gender expectations in either direction in any survey. Males, on the other hand, continue to show a significant bias towards males in all 11 surveys; in each case the magnitude of the effect declined, but remains significant. Fig 2 shows the consequences of this inequity by simulating the nominations that would occur according to this model in a hypothetical classroom with a 1:1 gender ratio and equal mean class performance and outspokenness by gender.

Another way to understand the magnitude of the gender bias is to compare its coefficient to that for class grade point average (GPA), our best proxy for actual mastery of course material scored on a 4 point scale. Averaged across the 11 surveys, females give a boost to fellow females relative to males that is equivalent to an increase in GPA of 0.040; i.e. they would be equally likely to nominate an outspoken female with a 3.00 and an outspoken male with a 3.04. On the other hand, males give a boost to fellow males that is equivalent to a GPA increase of 0.765; for an outspoken female to be nominated by males at the same level as an outspoken male her performance would need to be over three-quarters of a GPA point higher than the male’s. On this scale, the male nominators’ gender bias is 19 times the size of the female nominators’.

### Being male is a prerequisite for celebrity status

**Fig 3:**
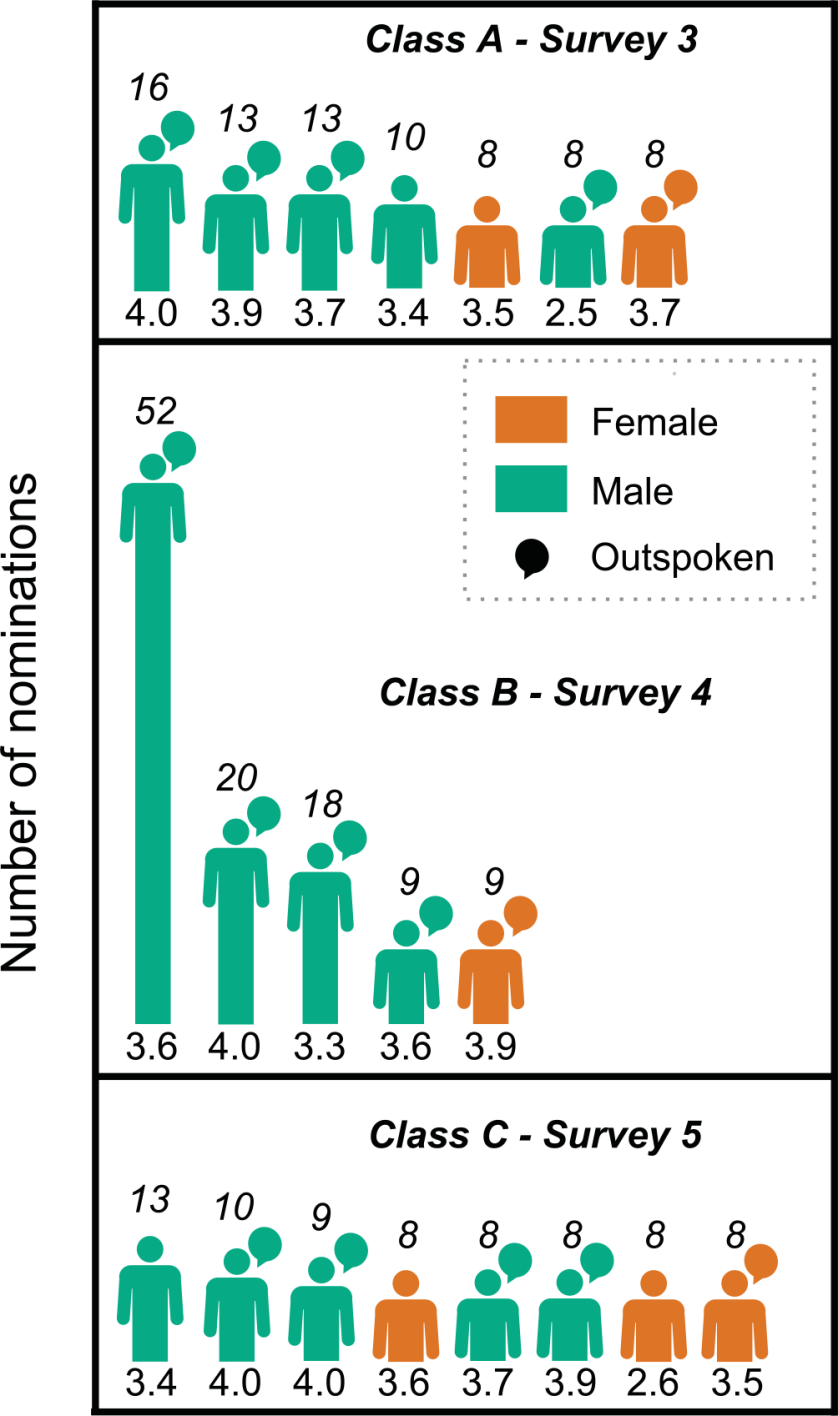
The most renowned students in each class tend to be male. Students with the five highest numbers of nominations are depicted for each class. The numbers above each student represent how many nominations that student received, while the numbers below each student represent their grade point average earned in the course out of 4 points. These data come from the last surveys administered in Classes A, B, and C, and represent our best estimate for the perceptions developed by the end of each class.

The three-to-four most nominated students in all classes examined were male. In each class, most students received very few nominations, while several students emerged over the course of the class as exceptionally well known; we refer to these students as “celebrities”. Several patterns are evident in the distribution of nominations in these classes (Fig 3). First, celebrity students tend to have high grades and speak up frequently in class. Second, with no exceptions, the biggest celebrity students in each network are male. While some females rank towards the top, the most well-known females are tied for 4^th^ in two classes, and are 5^th^ most well-known in the other. Third, male students at the top of the distribution tend to be considerably more well-known than any other student in the course. This is especially pronounced in Class B, where the most renowned male (52 nominations) received 5.78 times the nominations as the most renowned female (9 nominations). The most renowned male in Class A (16 nominations) has twice as many nominations than the most renowned female (8 nominations), while in Class C the most renowned male (13 nominations) has 1.63 times as many nominations than the most renowned female (8 nominations). These high nomination counts are notable, given the low average number of nominations seen across all 11 surveys (1.20).While the number of nominations achieved by celebrities in each class varies, the male biased pattern among the most frequently nominated peers holds.

The male majority among classroom celebrities could be explained if males were the only students who both achieved high grades and spoke up frequently in class. However, this is not the case. While male students on average scored slightly higher than female students and were more likely to be outspoken in every class, outspoken females with grades as high as these most renowned male students exist in every class (S4, S5 and S6 Figs). However, females achieving high grades and outspoken status never gain the same celebrity status as their male counterparts. It appears that being male is a prerequisite for students to achieve celebrity status within these classrooms.

## Discussion

The underrepresentation of women in STEM is a complex and daunting problem. Increasing gender equity requires tackling both inequalities in students’ initial interest in STEM and the retention of women who have expressed that interest. While there is strong evidence that precollege factors influence a student’s initial decision to major in a STEM field [24], the causes of attrition after students initially declare a STEM major are less commonly explored. Studies on attrition of STEM-oriented women have found sense of belonging [16], decisions to start families [24], and confidence that one can succeed in one’s chosen profession (11) all influence a woman’s decision to leave STEM. In particular, professional confidence is lower for women in STEM than for men [12]. This confidence is influenced by many inputs, but one of the major ones, especially for women in STEM fields, is receiving verbal messages and encouragement from individuals with influence, such as teachers and peers [9]. Unfortunately, current faculty hold a gender bias that impacts the experiences of women in STEM [13] and could result in less support from faculty. Here we demonstrate that the peers of female students in introductory biology classes can also exhibit gender biases, adding to the list of subtle experiences that can lead to the attrition of females from STEM careers.

In three iterations of an undergraduate biology class, we found that even after controlling for actual course performance and outspokenness, male peers still disproportionately nominate males as being knowledgeable about biology while females nominate males and females equally. This indicates that males hold a bias against their female peers’ competence in biology. Our finding of peers as a second source of differential treatment by gender, beyond known biases of faculty, contributes to a more complete picture of the experiences of undergraduate women in STEM fields. The coalescence of subtle messages about their STEM abilities from both faculty and peers may undermine the self-confidence females have to persist in STEM fields beyond their undergraduate education [25].

The finding that a gender bias impacts the perception of millennial students may at first seem surprising, but is supported by work on implicit biases. Implicit biases are unconscious associations that people hold related to certain groups. Across many cultures, STEM is associated with males and not females [26]. Interestingly, male STEM majors in the US hold the strongest associations between maleness and science, while female STEM majors show some of the weakest implicit biases between gender and science [27]. These differences in the gender-STEM stereotypes held may explain why male undergraduate STEM majors nominate more males, but females do not demonstrate this bias. It also helps explain why male faculty demonstrate biases in hiring and mentoring, but many female STEM faculty do not [28].

One potential analytical concern for the current study is multiple comparisons. This occurs when statistical analyses involves multiple outcome measures, testing for an effect of multiple independent variables on a single outcome measure, or when the research design is repeated across several populations. In each case, the chance of finding a false positive is increased by adding another test. Because we repeated our study design three times and include multiple independent variables in our models, we are performing multiple tests, and thus have increased chances of a false positive. However, the repeated significance of our main result (that males over-nominate their male peers) across every survey gives us no reason to suspect that they are spurious due to multiple comparisons. It appears that males consistently hold a bias against their female peers’ competence in biology.

### The classroom environment can influence student perceptions of their peers

Our work suggests that processes in the classroom may either be reinforcing pre-existing implicit biases over the quarter, or at least facilitating behaviors based on these biases. The end of every class term shows a stronger male bias than the beginning. This pattern is mediated by two class-related factors: 1) whether or not a student is outspoken in class and 2) level of achievement in the class. These factors, which seem to influence the opinions of both male and female peers, have previously been found to differ by gender in biology: males are more likely to be heard speaking in class and males slightly, but systematically, outperform women [15]. Instructors may be able to interrupt this process by equalizing the rate at which students of all genders speak up in class, closing the achievement gaps in their classrooms, or using more student-centered instruction in ways that do not rely primarily on whole class discussions (e.g. small group-work only).

We propose that the specific classroom environment can influence the effect size of the male bias, with some support for this hypothesis from Class C. In this term males did not behave differently than in previous years. Females, however, developed a stronger bias towards nominating other females than in the other two classes. Though this bias was not significant, it effectively lessens the overall magnitude of bias towards male students. Although we cannot specifically pinpoint why this was the case, this class differed from the other two in two critical ways. First, one of the three instructors in this course was female, whereas all instructors were male in the other two classes. Female instructors, when they are considered role models, have been shown to reduce the science-gender biases of female students, and this may have impacted the latter’s nomination patterns [29]. Second, during classroom discussions, all three instructors in Class C employed ‘random call,’ in which the instructor selects students to speak based on a randomized class list rather than by choice, more extensively than in the two other classes. Random call has been shown to eliminate the gender gap in class volunteers, leading to more females speaking up in class [15] and limiting the opportunity for one student to dominate classroom conversations and the instructors attention [30]. These differences in Class C seem to indicate that other factors in the classroom environment could mitigate the extent to which gender and renown are correlated. It is important to keep in mind that this mitigation seems to come from a larger female-female bias. This counteracting gender bias is likely undesirable compared to eliminating the male-male bias and achieving complete gender equity. Further research is needed to understand how to best achieve this equity in peer perception.

### Biology is a conservative case; patterns may be more extreme across STEM

The context of this research on peer perceptions was an introductory biology classroom. We can only speculate on the peer biases present in other STEM fields, but we predict that the male bias observed in this study may be conservative relative to other STEM fields for three reasons. First, biology is thought to be the STEM field with the most gender equity: undergraduate enrollment is nearly equal in terms of males and females [31] and slightly more women than men earn degrees in the biological sciences. Thus females in biology do not have to contend with the biases associated with being the sole representative of their gender in a STEM classroom [32]. Second, there is also a perception that biology lacks a strong math basis, and does not invoke the math-gender stereotype as strongly [33]. Thus, stereotypes about women’s math ability may not be undermining how their peers perceive them to the same extent it might in more explicitly math-based fields like physics or computer science. Finally, biology is a field that people believe does not require “brilliance”, unlike other STEM fields [34]. This perception means that stereotypes that males are more intelligent may not impact peer perceptions as strongly as it does in fields that are considered to require brilliance, like physics and math. For these reasons, we argue that the gender inequities in peer perception in the classrooms presented in this paper are likely conservative compared to classrooms in other STEM fields. Further, this dynamic may exist beyond STEM fields. However, explicit tests are required to confirm these hypotheses.

## Conclusion

Our findings have strong implications regarding the effectiveness of existing strategies to increase women in STEM fields. Without addressing social dynamics that perpetuate gender biases in the college classroom, simply increasing the number of young women entering STEM majors may not be enough. The patterns of uneven peer perceptions by gender shown in our student population suggest that future populations of academics may perpetuate the same gender stereotypes that have been illuminated among current faculty. This may not only be the case because the male students receiving high celebrity are reaffirmed in their abilities and are better able to advance through the STEM pipeline than women who do not receive this affirmation, but also because the existence of “celebrity” males and other individuals with distinction can impact and reaffirm the stereotypes held by others [35]. This gender biased pattern in celebrity was experienced by over 1,500 students in our analyses. This number is striking, but less worrisome than the millions of students who attend college STEM classes that may perpetuate the same biases described here. In addition to current impacts on the peers in their classes, the students in these classes are potential future faculty members. Although we cannot directly compare the magnitude of gender bias between current faculty and millennial students, our work implies that the chilly environment for women may not be going away any time soon.

## Supporting Information

S1 Appendix Further information about data collection and analyses. (DOCX)

S1 Fig Sociographs from all surveys in Class A. Male students are represented by green circles and females by orange circles. The size of nodes correlates with how many nominations each student received in the corresponding survey. Arrows show direction from the nominator to the nominee.
(EPS)

S2 Fig Sociographs from all surveys in Class B. Male students are represented by green circles and females by orange circles. The size of nodes correlates with how many nominations each student received in the corresponding survey. Arrows show direction from the nominator to the nominee.
(EPS)

S3 Fig Sociographs from all surveys in Class C. Male students are represented by green circles and females by orange circles. The size of nodes correlates with how many nominations each student received in the corresponding survey. Arrows show direction from the nominator to the nominee.
(EPS)

S4 Fig Plot showing outspoken students who scored in the top 10% of the class and nominations earned at the last survey collection in Class A. Even though outspoken females with extremely high scores exist, they fail to reach the same “celebrity” status as their male counterparts.
(TIFF)

S5 Fig Plot showing outspoken students who scored in the top 10% of the class and nominations earned at the last survey collection in Class B. Even though outspoken females with extremely high scores exist, they fail to reach the same “celebrity” status as their male counterparts.
(TIFF)

S6 Fig Plot showing outspoken students who scored in the top 10% of the class and nominations earned at the last survey collection in Class C. Even though outspoken females with extremely high scores exist, they fail to reach the same “celebrity” status as their male counterparts.
(TIFF)

S7 Fig Goodness of fit diagnostics for full ERGM model. Plots compare the in-degree distribution across students in the observed data to that for 10 network simulations from the model. Plots cover the six networks from the first two classes in consecutive order (top row: Course A, S2 and S3; middle row: Course B, S1 and S2; bottom row: Course B, S3 and S4). The x-axis is defined by number of nominations (“in-degree”), and the y-axis by the proportion of students displaying that in-degree. Thick black lines represent the observed distribution. Boxplots represent the simulations, with boxes representing the median and interquartile range, whiskers representing the minimum and maximum, and circles and gray lines representing the 95% support intervals.
(TIFF)

S8 Fig Plots showing the correlation between exam scores and GPA (Course grade on a 4.0 scale). Data points are represented as numbers (1–4) and colors (black, red, green, and blue) corresponding to the first, second, third, and fourth exams. In each class, exam scores correlate strongly with overall course grades. Due to this correlation, we chose to simplify our analyses by using course grade as a predictor across all models as opposed to using a unique contemporaneous exam scores at each time point.
(PNG)

S1 Table Model 1 shows ERGM results showing the effect of gender on the likelihood of a nomination for all 11 networks. This model controls for mutuality, and thus takes into account the increased likelihood of a nomination from student A to student B, given a nomination from B to A. This model shows the gender bias in nominations before taking into account outspokenness and class performance.
(DOCX)
