# Supplementary material for: Males Under-Estimate Academic Performance of Their Female Peers in Undergraduate Biology Classrooms
Source: PLoS One. 2016 Feb 10;11(2):e0148405. doi: 10.1371/journal.pone.0148405 (PMC4749286; doi:10.1371/journal.pone.0148405)
Supplement: S1 Appendix — (DOCX) [file pone.0148405.s001.docx]

**S1 Appendix**

**Data collection**:

Data collections methods varied slightly between the three classes. The data collection method for Classes A and B makes it impossible to determine retroactively whether students failed to complete the nomination form or purposefully chose not to nominate any students. This limitation was noted prior to Class C, and the form was modified to include an explicit “I chose not to nominate anyone” option. If a student chose the latter option, all of their out-ties were coded as 0’s; if they returned a blank form or did not return a form, they were coded as missing. The ergm framework includeds methods for conducting model estimation in the presence of missing tie information among a known set of actors (Gile and Handcock), which we employed for Class C. For Classes A and B, we coded all cases where a student provided no nominations in the 0. In order to determine the impact that this had on our analyses, we also analyzed the data from Class C with all of the missing data recoded as 0s.

**Use of course grades as predictor variables**:

Final course grades are used as dependent variables in ERGMs even though these grades are not created until after the prestige surveys were administered. The use of final grades in models creates continuity across the longitudinal models, which allows for a more straight forward interpretation of the results. The alternative would be to use students’ accumulated exam scores to the point of the survey. However, exam grades are highly correlated with final grades as shown in SI Figure S8, and interpretation of model results would be made more difficult. Further, when run with accumulated exam scores instead of final grades, ERGMs have the same results. For these reasons, final grade is used in all ERGMs instead of accumulated exam scores.

**Model fit**

Given the aims of our analysis, the most salient aspect of network structure is the distribution of in-degree across individuals; a model that did not capture the overall level of dispersion in this metric might make our inference on rates of nominations by the gender of recipient more or less conservative than is appropriate. To determine whether this was the case, we used the *gof* function within the *ergm* package, which simulates a set of networks from the given model, and compares them to the observed network on the characteristics of interest (Hunter et al 2008). We were able to do this for the six networks from the first two classes. The third class contained non-trivial amounts of missing data representing students who did not fill out the questionnaires, but were available to be nominated, as explained in the body of the manuscript. This is not a problem for model estimation itself, since *ergm* includes methods for recognizing missing data and fitting the model conditional on them (Handcock and Gile 2010). However, it does preclude traditional goodness-of-fit methods, which simulate over the missing data, effectively imputing their likely values, and thus the summary measures that emerge from these networks are no longer directly comparable to the analogous summary measures in the observed data. Nevertheless, we have no reason to believe that the results that hold consistently across the first six networks would be systematically different across the latter five.

Our initial models did not include the *0-indegree* term. Conducting goodness of fit analyses on these indicated that the models were indeed doing a poor job of capturing the overall shape of the in-degree distribution; the networks predicted by the model consistently had too few individuals with no nominations, too many with a few, and too few with a moderate to high number; that is, they were underdispersed. Given this, we elected to add in an additional term to capture the added propensity for no nominations. This single statistic allowed us to do a far superior job of capturing the full distribution while only using up a single degree of freedom. Figure S7 shows the goodness of fit plots for in-degree for the six networks without missing data. We see that the general shape of the distribution is well captured by our model, with the observed frequencies in or near the central 95% of the range of variation of the simulations in most cases. Our model does not capture every detail of the distribution, including the exact locations at which the right-hand outliers occur; however, we considered that adding additional terms would make the model more dificult to interpret and risk overfitting.

**References:**

Handcock MS and Gile KJ. 2010. Modeling social networks from sampled data. *Annals of Applied Statistics*. 4(1): 5-25.

Hunter DR, Goodreau SM, and Handcock MS**.** 2008. Goodness-of-fit of social network models. *Journal of the American Statistical Association*. 103(481): 248-258.
