# Supplementary figures and images for: Males Under-Estimate Academic Performance of Their Female Peers in Undergraduate Biology Classrooms
Source: PLoS One. 2016 Feb 10;11(2):e0148405. doi: 10.1371/journal.pone.0148405 (PMC4749286; doi:10.1371/journal.pone.0148405)

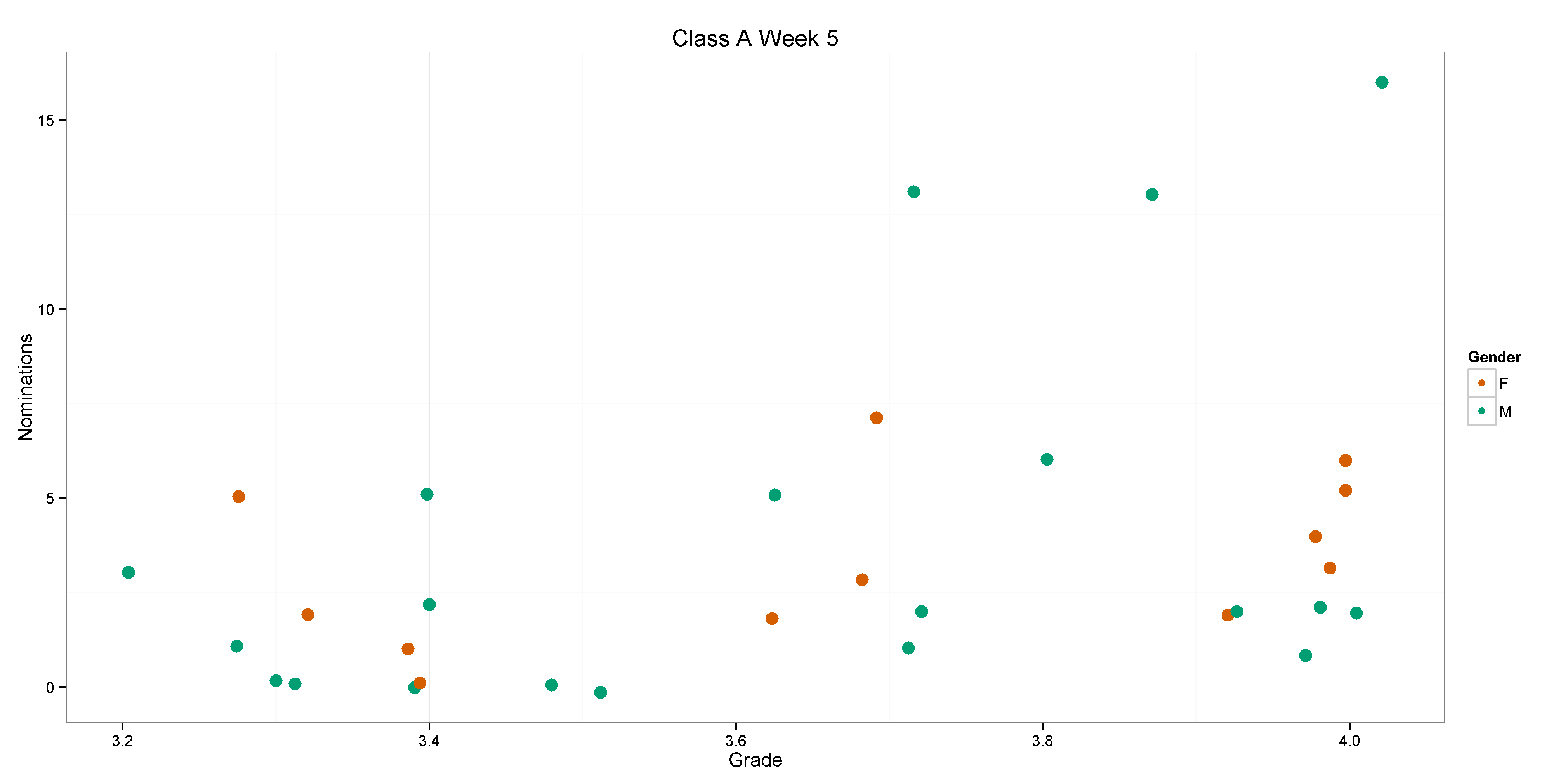

Supplement: S4 Fig — Even though outspoken females with extremely high scores exist, they fail to reach the same “celebrity” status as their male counterparts. (TIFF) [file pone.0148405.s005.tiff]

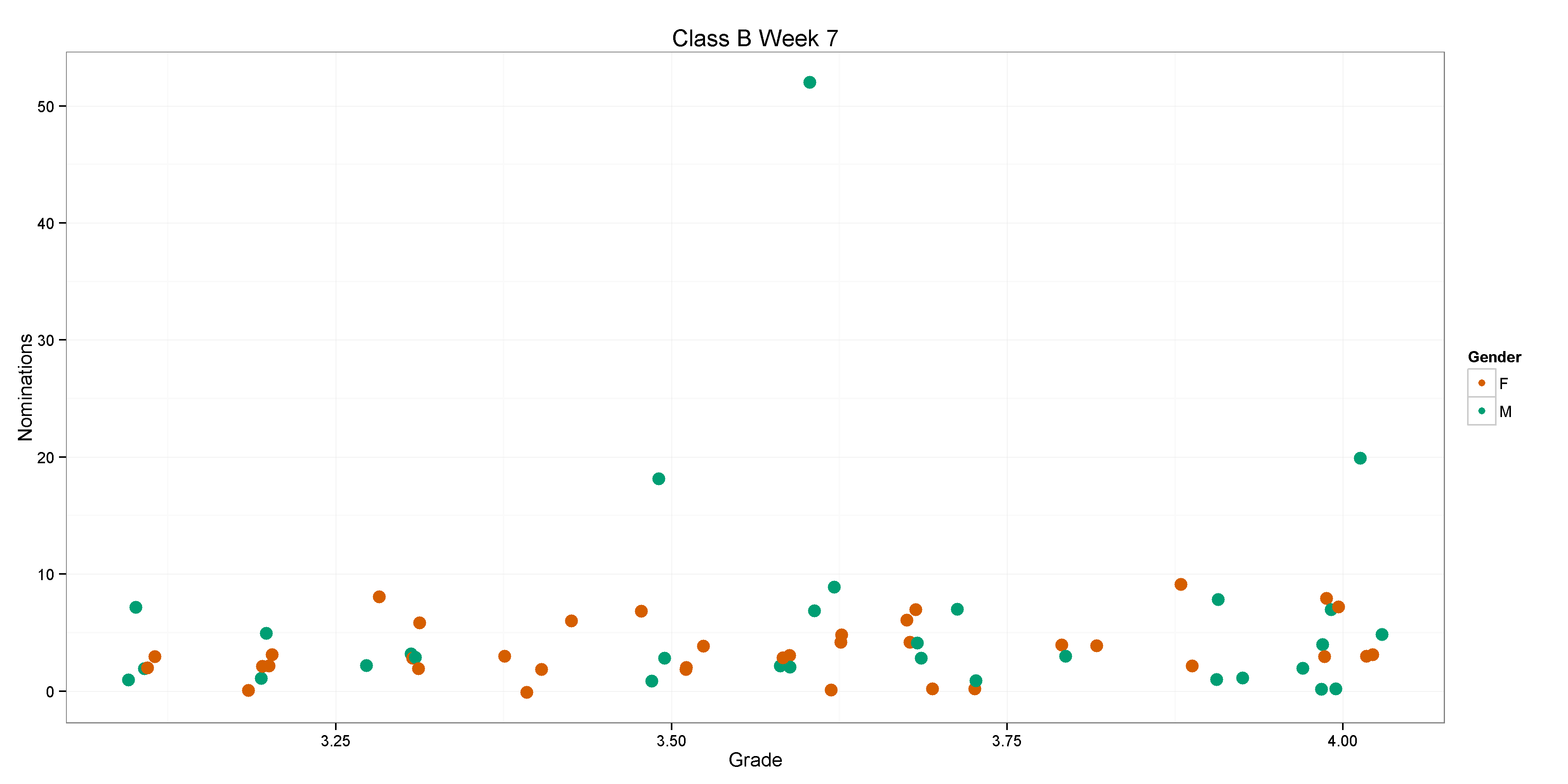

Supplement: S5 Fig — Even though outspoken females with extremely high scores exist, they fail to reach the same “celebrity” status as their male counterparts. (TIFF) [file pone.0148405.s006.tiff]

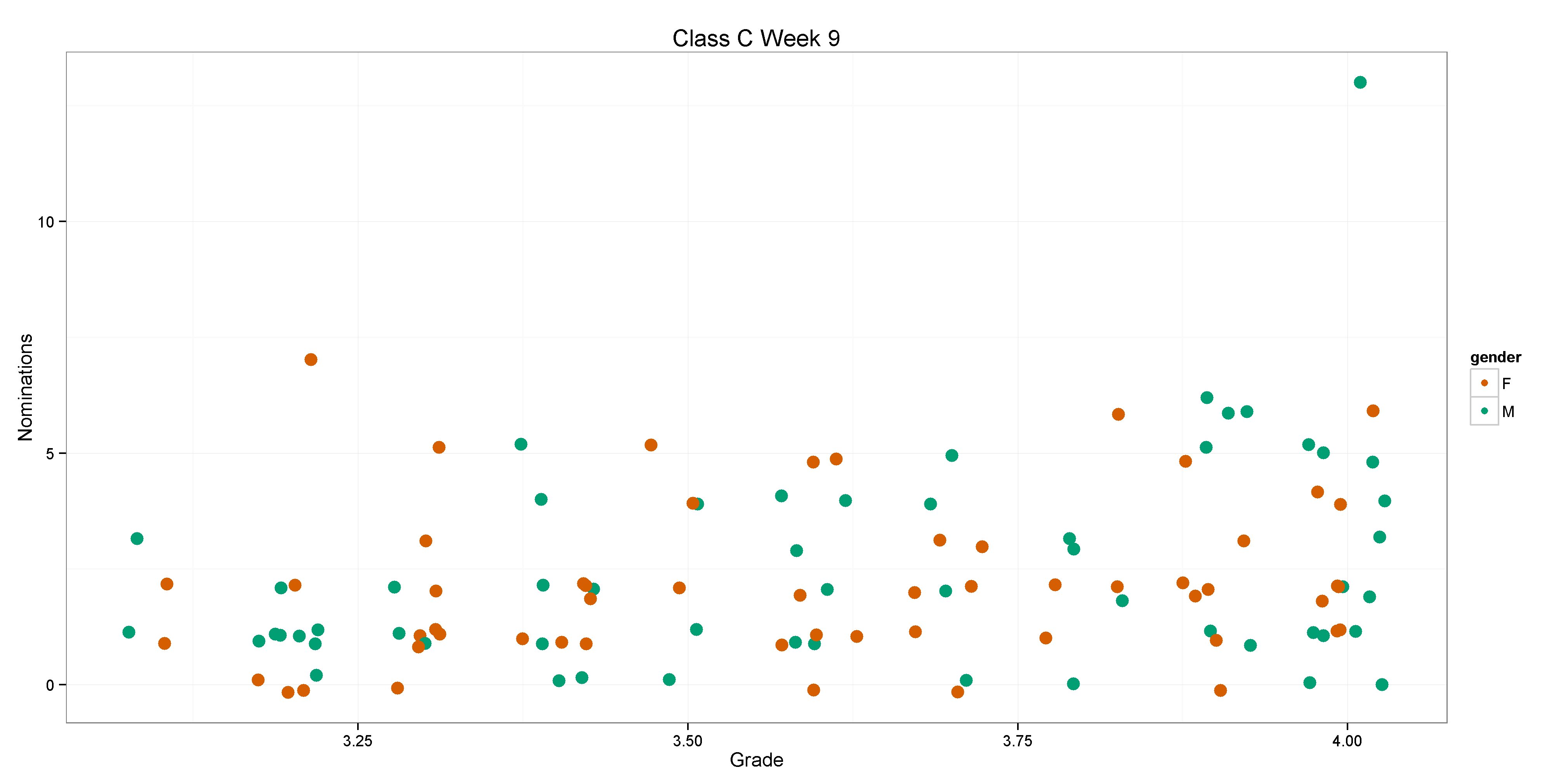

Supplement: S6 Fig — Even though outspoken females with extremely high scores exist, they fail to reach the same “celebrity” status as their male counterparts. (TIFF) [file pone.0148405.s007.tiff]

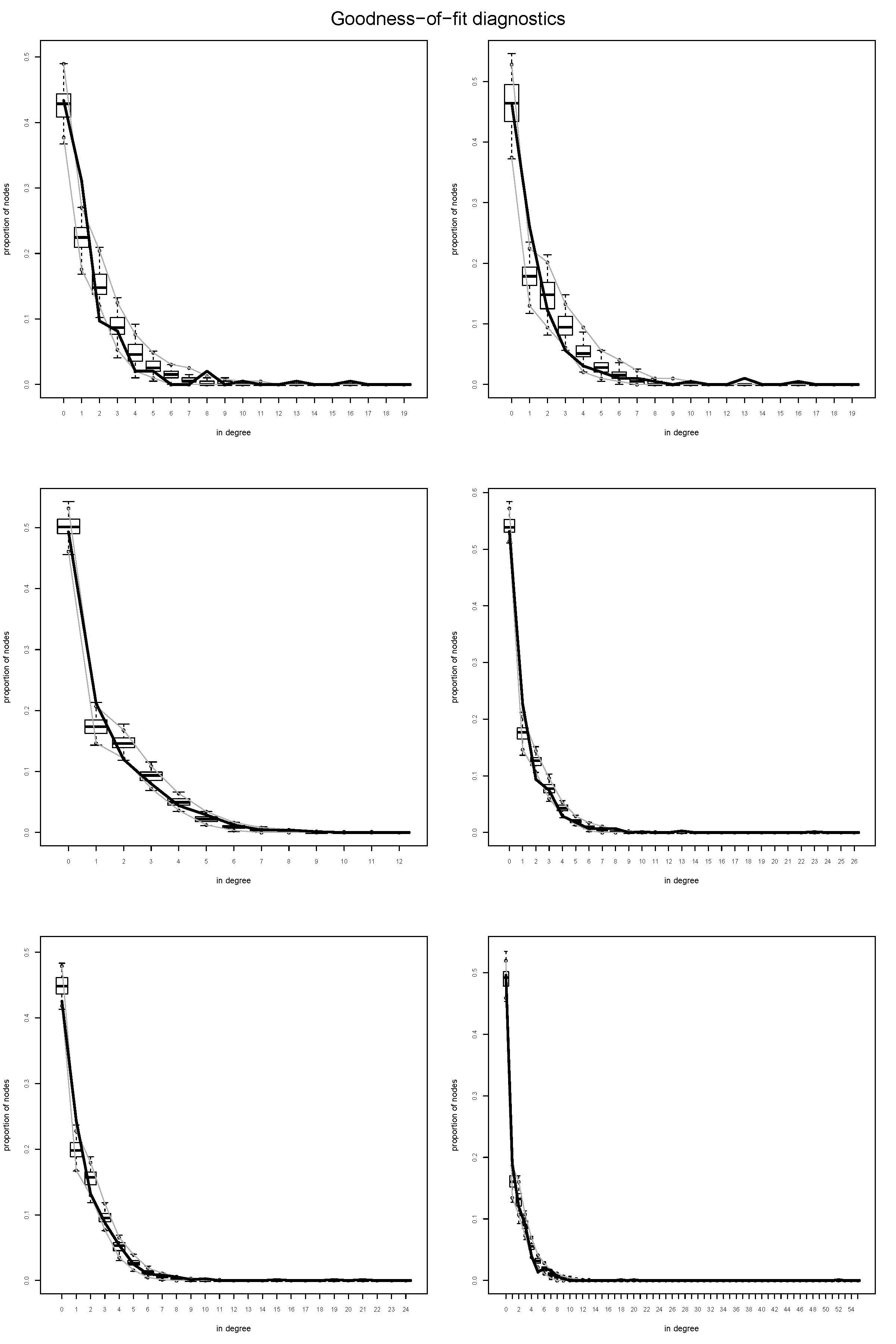

Supplement: S7 Fig — Plots compare the in-degree distribution across students in the observed data to that for 10 network simulations from the model. Plots cover the six networks from the first two classes in consecutive order (top row: Course A, S2 and S3; middle row: Course B, S1 and S2; bottom row: Course B, S3 and S4). The x-axis is defined by number of nominations (“in-degree”), and the y-axis by the proportion of students displaying that in-degree. Thick black lines represent the observed distribution. Boxplots represent the simulations, with boxes representing the median and interquartile range, whiskers representing the minimum and maximum, and circles and gray lines representing the 95% support intervals. (TIFF) [file pone.0148405.s008.tiff]

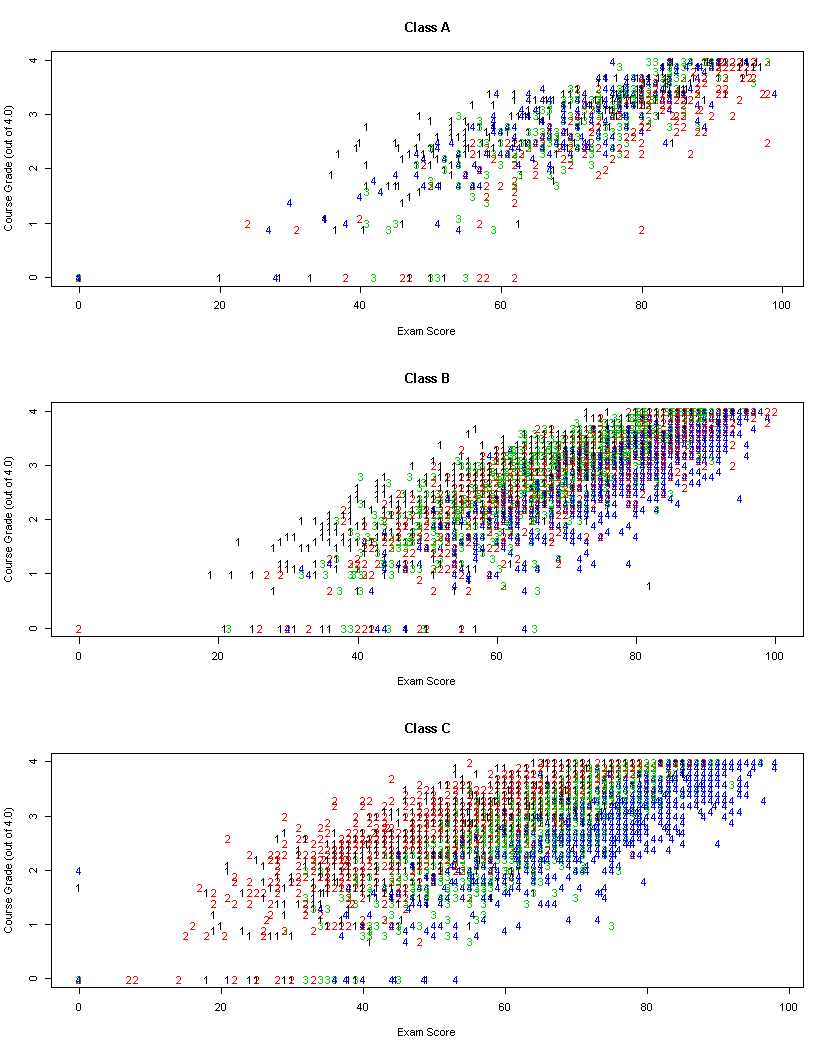

Supplement: S8 Fig — Data points are represented as numbers (1–4) and colors (black, red, green, and blue) corresponding to the first, second, third, and fourth exams. In each class, exam scores correlate strongly with overall course grades. Due to this correlation, we chose to simplify our analyses by using course grade as a predictor across all models as opposed to using a unique contemporaneous exam scores at each time point. (PNG) [file pone.0148405.s009.png]
