# Supplementary material for: Males Under-Estimate Academic Performance of Their Female Peers in Undergraduate Biology Classrooms
Source: PLoS One. 2016 Feb 10;11(2):e0148405. doi: 10.1371/journal.pone.0148405 (PMC4749286; doi:10.1371/journal.pone.0148405)
Supplement: Supplementary file 3 [file pone.0148405.s010.docx]

Coefficient name	Course A		Course B				Course C				
	S2	S3	S1	S2	S3	S4	S1	S2	S3	S4	S5
Intercept	**-5.83 (-6.22, -5.43)**	**-5.79 (-6.20, -5.39)**	**-6.70 (-6.88, -6.52)**	**-6.65 (-6.84, -6.45)**	**-6.77 (-6.94, -6.60)**	**-6.65 (-6.84, -6.47)**	**-6.57 (-6.75, -6.38)**	**-6.56 (-6.74, -6.38)**	**-6.53 (-6.72, -6.35)**	**-6.40 (-6.57, -6.22)**	**-6.50 (-6.68, -6.32)**
Mutuality	**3.65 (3.16, 4.14)**	**4.38 (3.90, 4.85)**	**5.49 (5.19, 5.80)**	**5.62 (5.30, 5.93)**	**5.84 (5.60, 6.08)**	**5.42 (5.17, 5.66)**	5.76 (5.49, 6.03)	**5.37 (5.10, 5.64)**	**5.46 (5.17, 5.75)**	**5.28 (5.04, 5.52)**	**5.47 (5.21, 5.73)**
Homophily on lab section	**1.44 (1.20, 1.68)**	**1.07 (0.84, 1.31)**	**0.71 (0.53, 0.90)**	**1.07 (0.88, 1.25)**	**1.19 (1.05, 1.34)**	**1.22 (1.07, 1.37)**	**1.10 (0.92, 1.29)**	**1.20 (1.01, 1.40)**	**1.33 (1.07, 1.59)**	**1.40 (1.22, 1.58)**	**1.34 (1.16, 1.51)**
0-indegree	**1.52 (1.08, 1.97)**	**1.93 (1.47, 2.39)**	**1.81 (1.57, 2.04)**	**1.94 (1.69, 2.20)**	**1.71 (1.48, 1.95)**	**2.37 (2.13, 2.61)**	**1.69 (1.41, 1.97)**	**1.64 (1.36, 1.91)**	**1.90 (1.62, 2.18)**	**1.78 (1.50, 2.05)**	**1.69 (1.42, 1.96)**
Female nominator	**0.97 (0.53, 1.41)**	**1.22 (0.77, 1.67)**	**0.62 (0.40, 0.84)**	**0.50 (0.28, 0.72)**	**0.60 (0.39, 0.81)**	**0.77 (0.56, 0.98)**	**0.35 (0.10, 0.59)**	**0.32 (0.09, 0.55)**	**0.36 (0.14, 0.59)**	0.24 (0.02, 0.45)	**0.29 (0.06, 0.51)**
Female-female bias	-0.19 (-0.45, 0.07)	**-0.35 (-0.60, -0.11)**	0.02 (-0.12, 0.17)	-0.05 (-0.19, 0.10)	-0.12 (-0.26, 0.03)	-0.14 (-0.28, 0.01)	0.08 (-0.08, 0.24)	0.12 (-0.03, 0.26)	0.07 (-0.09, 0.22)	0.07 (-0.08, 0.23)	0.09 (-0.05, 0.23)
Male-male bias	**0.69 (0.29, 1.09)**	**0.67 (0.27, 1.06)**	**0.40 (0.21, 0.58)**	**0.31 (0.10, 0.53)**	**0.42 (0.22, 0.62)**	**0.56 (0.37, 0.76)**	**0.33 (0.11, 0.55)**	**0.36 (0.17, 0.56)**	**0.36 (0.16, 0.57)**	**0.39 (0.20, 0.58)**	**0.35 (0.15, 0.54)**

Cell entry = Point estimate (95% CI); S = survey number; Bold = signficant ($p < 0.05$)
